# Therapeutic Efficacy of Nasoenteric Tube Feeding in Children Needing Enteral Nutrition

**DOI:** 10.3389/fped.2021.646395

**Published:** 2021-03-18

**Authors:** Mi-Chi Chen, Hsun-Chin Chao, Pai-Jui Yeh, Ming-Wei Lai, Chien-Chang Chen

**Affiliations:** ^1^Division of Pediatric Gastroenterology, Department of Pediatrics, Chang Gung Children's Medical Center, Chang Gung Memorial Hospital, Taoyuan City, Taiwan; ^2^Chang Gung University College of Medicine, Taoyuan City, Taiwan

**Keywords:** children, enteral nutrition, naso-enteric tubes, nutritional status, gastrointestinal reflux

## Abstract

**Background:** There is limited information on therapeutic benefits and tube-related complications of pediatric nasoenteric (NE) tube feeding. We viewed, from different clinical aspects, NE tube feeding in children who are under intolerable conditions.

**Methods:** A 10-years retrospective study enrolled 77 pediatric patients who underwent an endoscopic-guided placement of the NE tube for enteral nutrition. The evaluated data, including growth parameters, feeding volume, parenteral nutrition (PN) dependence, and nutritional markers [serum hemoglobin (Hb) and albumin] before and after NE tube feeding were compared. Tube-related complications and major adverse events were also recorded.

**Results:** A total of 77 patients (including 50 males) underwent 176 endoscopic-guided placements of the NE tube with an average duration of 133.7 (6.0–1,847.3) days. The gastroesophageal reflux disease-related symptoms (vomiting, desaturations, and aspiration pneumonia) improved in 71.4% of patients. Feeding volume increased significantly after intervention, especially in patients with delayed gastric emptying, from 144.8 ± 28.5 to 1,103.1 ± 524.7 ml/days (*p* < 0.001). Weaning from PN was successfully achieved in 84.3% of patients with an average of 9.33 ± 7.30 days. About 16 patients (20.8%) were subsequently highly compatible with oral feeding after NE tube placement for an average of 24.7 ± 14.1 days. Patients either without neurologic dysfunction or with no ventilator-dependent status had a higher chance of shifting to oral feeding. Weight-for-age *z*-scores increased by 0.15 ± 1.33 after NE tube intervention. One NE tube-related adverse event, which caused bowel perforation at 6 days post-insertion, was recorded. No direct tube-related mortality was observed.

**Conclusions:** Endoscopic-guided NE tube placement is a relatively safe, non-invasive procedure for pediatric patients who require enteral nutrition. Feeding *via* NE tube showed beneficial effects such as improvement in symptoms, PN weaning, and maintenance of body growth without major tube-related complications.

## Introduction

Nutritional support is indicated for patients with inadequate nutrition intake or manifestation of wasting and stunting (1). Enteral feeding is more favorable than parenteral feeding in patients with a functioning digestive tract because the former can maintain gut integrity and prevent bacterial translocation (2). Different tube feeding strategies are available for short- or long-term use. In general, nasogastric (NG) tube is the most common and easiest route for pediatric patients to provide nutrition support (3).

Enteral tube feeding involves the artificial delivery of nutrition directly to the gastrointestinal tract without the need for swallowing. In temporary or short-term situations, this method is most commonly performed *via* an NG tube into the stomach but can be achieved *via* post-pyloric access with a nasoenteric (NE) tube (nasoduodenal or nasojejunal tube) into the proximal small bowel (4). Post-pyloric access is indicated in specific situations, such as severe gastrointestinal reflux disease (GERD) with a risk of aspiration, gastric emptying dysfunction, gastric outlet obstruction, acute pancreatitis, and previous gastric surgery precluding gastric feeding or in early postoperative feeding after major abdominal surgery (1, 5, 6). The common candidates for NE tube feeding include neurological disability, critically ill children, and young infants (6).

Several studies established the efficacy of small bowel feeding for nutritional support, and growing evidence showed benefits for infants and children who fail to thrive under intragastric feeding (6, 7). The present study aims to investigate the clinical outcomes of pediatric patients who received enteral nutrition *via* endoscopic-guided NE tube feeding in a tertiary hospital.

## Methods

### Study Design

Children who were aged <18 years and who underwent endoscopic-guided NE tube placement for enteral nutrition between January 2011 and June 2020 were enrolled in the study. Four indications used for NE tube placement are described as follows: (i) severe GERD with recurrent emesis despite prokinetic and antacid treatment, recurrent aspiration pneumonia, or frequent desaturation or bradycardia attack during or after feeding; (ii) delayed gastric emptying with gastric residuals of over 50% of the administered volume in the previous 4 h (8); (iii) post-surgery nutritional support with a functioning gut but with complete intolerance to oral or NG tube feeding within the first postoperative week; and (iv) partial obstruction of the upper gastrointestinal tract (UGI) demonstrated by using barium study or direct endoscopic visualization. Except for contraindications to NG feeding, most of the enrolled patients tried NG feeding but experienced intolerance or were unable to meet the nutritional requirements to achieve adequate body growth. We suggest gastrostomy (GT) (with or without Nissen fundoplication) or jejunostomy for patients who require long-term enteral nutrition for more than 4–6 weeks (9–11) and for some of our patients who had prolonged use of the NE tube for feeding due to unsuitability to or family's refusal to GT or jejunostomy for long-term feeding.

Medical records, including demographic information, underlying diseases, nutritional status (body weight, body height/length, and BMI), laboratory data [Hb, mean corpuscular volume (MCV), and albumin], and average daily feeding volume (assessed by a 3-days dietary record), were collected. The laboratory data and feeding volume at the initiation and at the end of NE tube feeding were compared. The data on the evaluated serum albumin were obtained at 2–3 weeks before and 2–3 weeks after the end of NE tube feeding (12).

### The Procedure for Nasoenteric Tube Placement

In our institution, we offer endoscopic-guided NE tube placement for patients who are unable to tolerate NG tube feeding for variable periods. Unweighted polyurethane NE tubes (CORFLO Enteral Feeding Tubes) with sizes of 6–12 French based on age and body weight were provided to the patients. The patients were sedated using intravenous midazolam (0.1–0.2 mg/kg/dose), and their vital signs during the procedure were monitored. The NE tube was placed beyond the second portion of the duodenum through the nose under endoscopic guidance (5, 13). The injection and withdrawal of dis-water were tested for function assurance. X-ray confirmation of a proper tube location before the initiation of feeding is mandatory at our institution (10). Ideally, the tip position is located beyond the ligament of Treitz and proximal jejunum. The nutritionist was advised with a feeding formula and feeding schedule individually. The scheduled time for exchanging NE tubes was every 3 months or the time until tube dysfunction.

### Outcome Measures

The measured outcomes of NE tube feeding included nutritional promotion, successful replacement of parenteral nutrition (PN), improvement of symptoms, subsequent feeding strategy (oral, NG, and GT or jejunostomy) after discontinuation of NE tube feeding, and complications, such asdislodgement, occlusion, breaks, migration to incorrect position, refeeding syndrome, intussusception, bowel perforation, serious bacterial enterocolitis, and mortality). Nutritional promotion measurement included nutritional markers (Hb and serum albumin level) and nutritional status. The nutritional status was measured by using weight-for-age *z*-score and height-for-age *z*-score, calculated using WHO Anthro v.3.2.2 software (World Health Organization, Geneva, Switzerland) in patients who were younger than 5 years old and using WHO AnthroPlus v.1.0.4 software (WHO, Geneva, Switzerland) in patients aging 5–19 years old. The weaning of PN was defined as full enteral feeding without any additional intravenous fluids and PN support. The improvement of symptoms would be defined as an occurrence of vomiting <3 times per week without interfering with the advancement of feeding volume and vital sign changes (desaturations and bradycardia) at <1 time per week. A partial improvement is defined as a partial relief but not reaching the abovementioned goals. Tube-related complications would be reviewed from medical records.

### Statistical Analysis

The continuous variables are expressed as mean ± SD, median, and interquartile ranges if they followed a non-normal distribution. Comparison results were analyzed with the Student's *t*-test (normally distributed continuous variables), the Mann–Whitney U test (non-normal distributed variables), and the Chi-squared test (categorical variables). Status changes were analyzed with a paired *t*-test. Statistical analysis was performed by using Statistical Product and Service Solutions software version 24.0 (SPSS Inc., Chicago, IL, USA). A *p* < 0.05 was considered statistically significant.

### Ethical Approval

The study was approved by the Ethics Committee of the Chang Gung Memorial Hospital (Ref. 202001377B0).

## Results

### Patient Demographics

Between January 2011 and June 2020, 83 cases received endoscopic-guided NE tube placement at the Chang Gung Memorial Hospital. About five of the 83 eligible patients were excluded due to short-term NE tube placement, and one patient was excluded due to non-endoscopic guidance. Table 1 summarizes the characteristics of study patients. The mean age was 4.3 ± 5.41 years, and the age of 34 patients (44.2%) was <1 year. Exactly 49 patients (63.6%) had neurologic dysfunction, including cerebral palsy, encephalitis, brain tumor, intracranial hemorrhage, and neurodevelopmental delay. About 25 patients (32.5%) were born prematurely, and the average gestational age was 32.2 weeks (range: 26–36 weeks).

**Table 1:** Patient demographics in patients with nasoenteric (NE) tube feeding.

**Parameters**	**Values**
**Case number**	77
Age (years)	
Mean ± SD	4.30 ± 5.41
Median, IQR	1.24, 6.34
Male (%)	50 (64.9%)
**Body mass parameters**	
Weight (kg)	
Mean ± SD	13.59 ± 36.71
Median, IQR	8.4, 13.53
Weight-for-age *z*-score, mean ± SD	−2.75 ± 2.71
Length/ height (cm)	
Mean ± SD	86.47 ± 36.71
Median, IQR	75.5, 55
Height-for-age *z*-score, mean ± SD	−2.43 ± 2.70
**Medical conditions/comorbidity**	
Ventilator use^#^	40 (51.9%)
Cardiovascular dysfunction^*^	9 (11.7%)
Neurologic dysfunction	49 (63.6%)
Preterm	25 (32.5%)
Congenital abnormalities	14 (18.2%)
GI tract structural anomaly	10 (13%)
Burn	4 (5.2%)
Leukemia/Lymphoma	3 (3.9%)
Inborn errors of metabolism	2 (2.6%)

*SD, standard deviation; IQR, interquartile range*.

# *Ventilator use: under mechanical ventilation, including non-invasive ventilator*.

* *Cardiovascular dysfunction: under inotropes agent support, including epinephrine, dopamine, Milrinone*.

### Experiences of Endoscopic-Guided NE Tube Placement

In the study period, we performed 176 uneventful endoscopic-assisted NE tube placements. The success rate was 98.9%. The average duration of the placement of the NE tube was 133.7 days (range: 6–1,847). The most common indication was severe GERD (*N* = 42, 54.5%), followed by delayed gastric emptying (*N* = 23, 29.9%), partial UGI obstruction (*N* = 7, 9.1%), and postoperative nutritional support (*N* = 5, 6.5%). The GERD-related symptoms were vomiting refractory to medications (*N* = 31, 73.8%), desaturations/bradycardia during/after feeding (*N* = 15, 35.7%), aspiration pneumonia episode (*N* = 11, 26.2%), and choking (*N* = 10, 23.8%). The etiologies of patients with partial UGI obstruction (*N* = 7) included pylorus spasm (*N* = 2), antral web (*N* = 1), GT leakage (*N* = 1), trauma-related duodenal obstruction (*N* = 2), and superior mesenteric artery syndrome (*N* = 1). Four patients who required post-operation nutritional support (*N* = 5) were operated due to burn injuries, and one patient was operated due to necrotizing fasciitis.

### Therapeutic Efficacy of NE Tube in Place

#### (I) Symptom Improvement

In the severe GERD group (*N* = 42), 30 patients (71.4%) showed an obvious improvement and seven patients (16.7%) showed a partial improvement of vomiting and desaturations. NE tube feeding also helped to reduce the risk of aspiration pneumonia. Two patients with frequent aspiration pneumonia showed decreased episodes after NE tube feeding (the reduction of episodes from three times in 1 year to eight times in 5 years and from five times in 5 years to one time in 3 years), whereas the other patients who had a previous history of aspiration pneumonia experienced no recurrence during the NE tube feeding.

**Figure 1:**
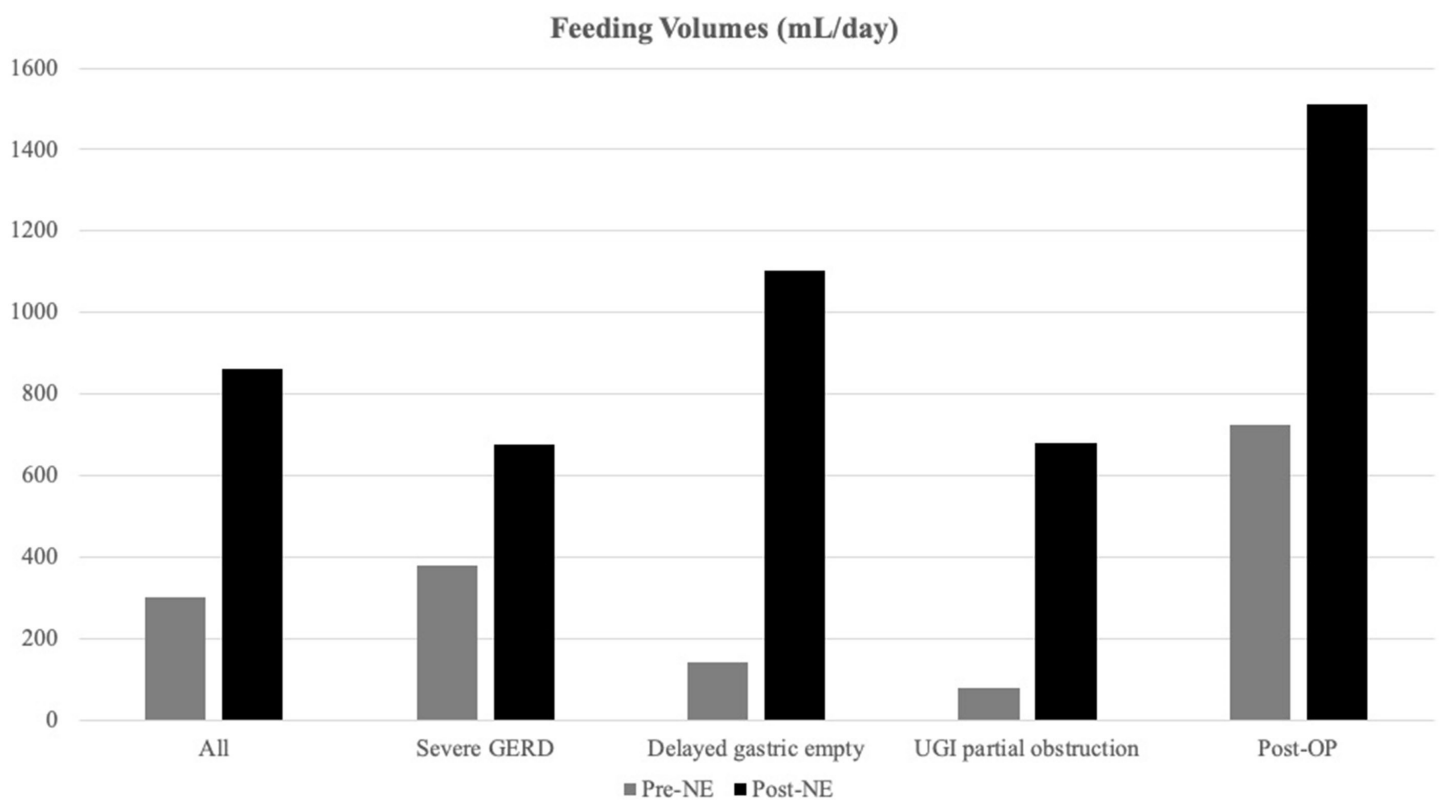
Feeding volume alternations after nasoenteric (NE) tube feeding. Feeding volumes were increased significantly after NE tube placement, especially in the delayed gastric emptying group (from 144.8 ± 136.5 to 1,103.1 ± 524.7 ml/days) than the non-delayed gastric emptying group from 373.5 ± 389.4 to 755.9 ± 451.3 ml/days, *p* < 0.0001).

Overall, the daily feeding volume significantly increased from 304.3 ± 349.0 to 861.0 ± 497.8 ml/days, *p* < 0.001 (Figure 1). In the delayed gastric emptying group, patients exhibited a significant increase in the daily volume from 144.8 ± 28.5 to 1,103.1 ± 524.7 ml/day, *p* < 0.001.

#### (II) Weaning From Parenteral Nutrition

About 32 patients (41.6%) required PN support before NE tube feeding. About 27 (84.3%) of 32 patients were weaned from PN successfully within 1 month with an average duration of 9.33 ± 7.30 days. Among PN-dependent patients (*N* = 5), two of them failed to achieve adequate feeding volume with NE tube feeding after 11 and 17 days, and the tubes were removed. One patient had NE tube-related bowel perforation, and one patient with total intestinal hypoganglionosis-related short bowel syndrome was dependent on PN. The fifth patient achieved full feeding but required a high protein intake due to severe burn injury.

#### (III) Nutritional Laboratory Markers

Serum Hb and albumin data of pre-NE and post-NE tubes in place were available for 67 and 35 patients, respectively. Mean pre- and post-NE tube Hb levels were 11.0 ± 1.7 g/dl and 11.3 ± 1.9 g/dl, respectively. The serum albumin level revealed a significant elevation from 3.22 ± 0.84 to 3.82 ± 0.67 g/dl after the intervention.

#### (IV) Growth Assessment

The average weight-for-age *z*-score for patients was −2.75 ± 2.71 and the average height-for-age *z*-score for patients was −2.43 ± 2.70. After NE tube placement, the patients exhibited an increment in weight-for-age *z*-score of value 0.15 ± 1.33 and in height-for-age *z*-score of value −0.27 ± 1.26 without statistical significance.

**Figure 2:**
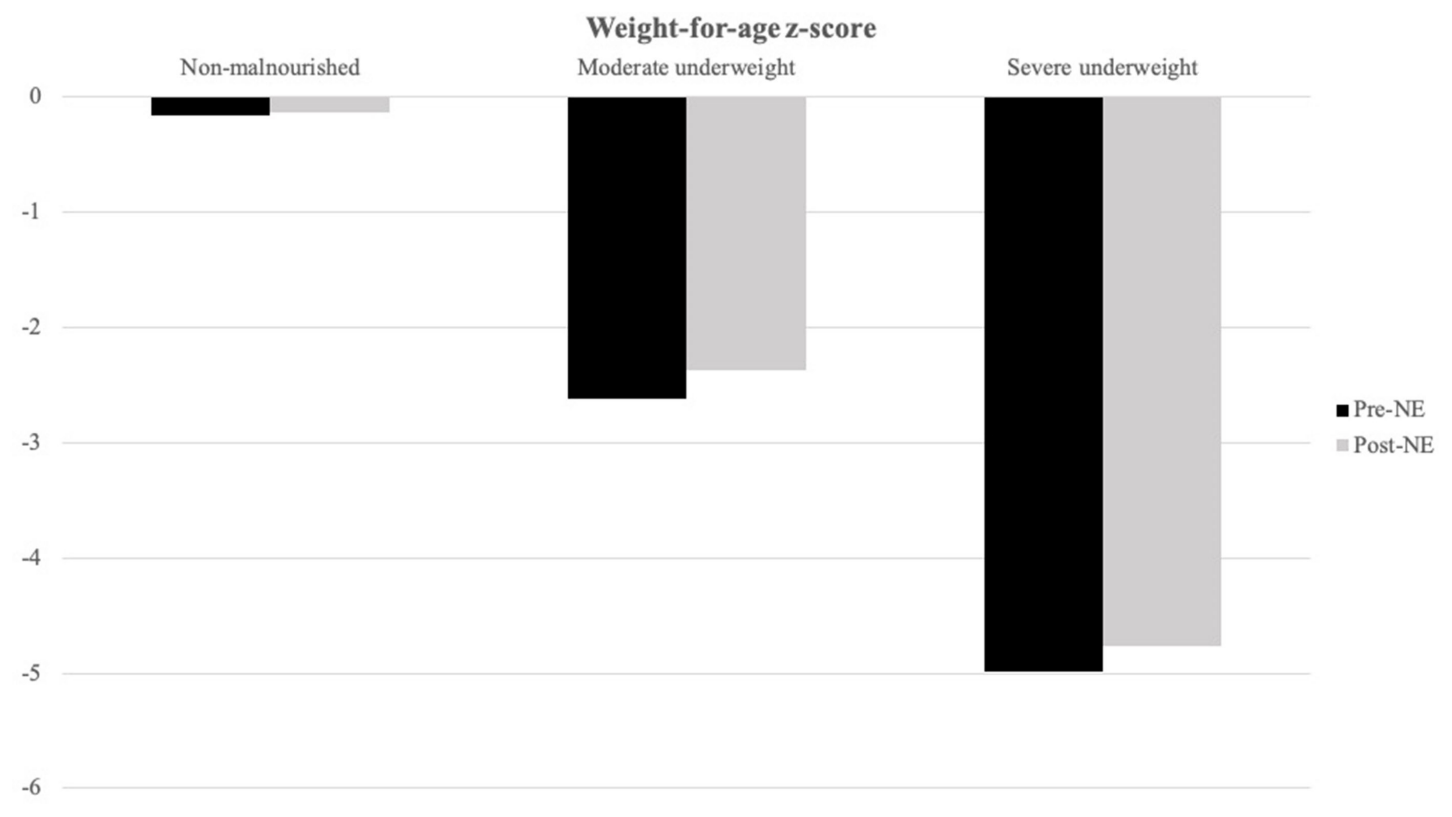
Weight-for-age *z*-score alternations after NE tube placement is stratified by baseline weight-for-age *z*-score. Moderate underweight (baseline weight-for-age *z*-score between −3 and −2, *N* = 8) and severe underweight group (baseline weight-for-age *z*-score below −3, *N* = 35) showed better weight-for-age *z*-score increment after NE tube placement than non-malnourished (baseline weight-for-age z-score above −2, *N* = 30) group patients.

Patients with severe underweight (weight-for-age *z*-score < −3) and severe stunting (height-for-age *z*-score < −3) accounted for 45.5 and 26.0%, respectively. We stratified patients with baseline weight-for-age *z*-score for three groups (Figure 2): non-malnourished (*z*-score more than −2, *N* = 30), moderate underweight (*z*-score between −3 and −2, *N* = 8), and severe underweight (*z*-score < −3, *N* = 35), and weight-for-age *z*-score increments for the three groups were 0.03 ± 1.33 (*p* = 0.889), 0.25 ± 0.62 (*p* = 0.292), and 0.23 ± 1.47 (*p* = 0.361), respectively.

**Figure 3:**
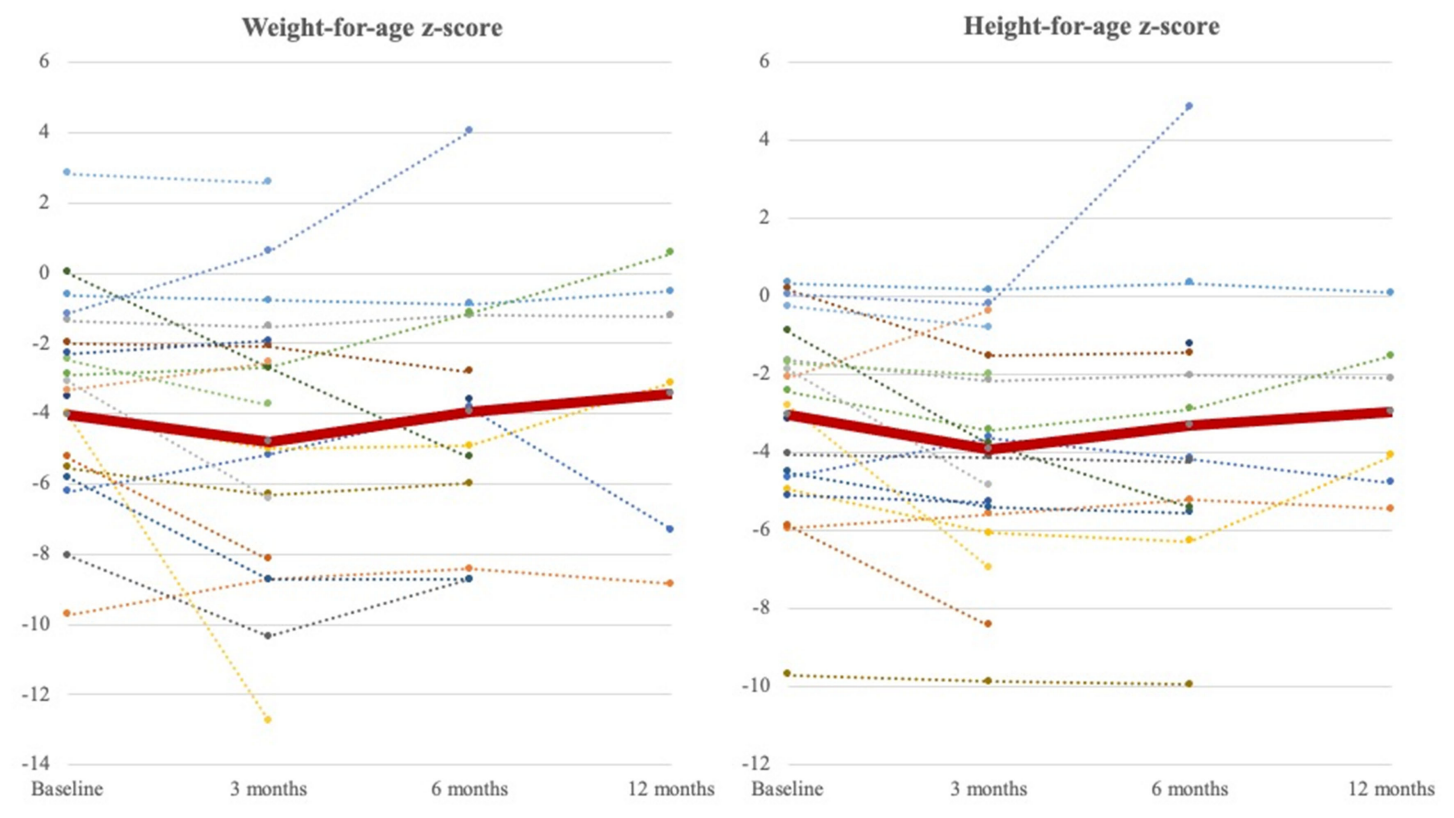
Weight-for-age *z*-score and height-for-age *z*-score curve at 3 months, 6 months, and 1 year during follow-ups. The dot lines represent weight-for-age *z*-score and height-for-age *z*-score of individuals (baseline: *N* = 20, 3 months; *N* = 20, 6 months; *N* = 13, 1 year: *N* = 6). The thick red line represents the average of all patients. The nadir occurred at 3 months after NE tube placement and increased to exceed baseline until a 1-year follow-up.

We further analyzed the growth of patients who had prolonged use of NE tube feeding for over 3 months (Figure 3). The average weight-for-age *z*-score and height-for-age *z*-score increased from −4.02 ± 2.87 to −3.42 ± 3.84 and from −3.05 ± 2.56 to −2.97 ± 2.13, respectively, in 1 year.

### Subsequent Feeding Strategy

Subsequent feeding strategiesy are summarized in Table 2. Overall, 16 patients (20.8%) experienced the successful removal of the NE tubes and tolerated oral feeding well with an average of 24.7 ± 14.1 days (range: 6–68 days). Patients without neurologic dysfunction had a higher rate of changing to oral feeding than those with neurologic dysfunction (41.7 vs. 6.1%, *p* < 0.001). Patients who were initially under ventilator support had a lower chance of shift to oral feeding than those without the support (2.6 vs. 35.3%, *p* < 0.001).

**Table 2:** Clinical outcomes of NE-tube placement.

	**All**	**Severe GERD**	**Delayed gastric empty**	**UGI partial obstruction**	**Post-OP**
Number	77	42	23	7	5
NE duration (days)^*^	133.7 ± 232.9	161.9 ± 342.7	117.3 ± 170.1	96.6 ± 128.4	27.2 ± 4.0
**Subsequent feeding strategy**					
Oral feeding	16 (20.8%)	6 (14.3%)	2 (8.7%)	4 (57.1%)	4 (80.0%)
NG	30 (39.0%)	15 (35.7%)	13 (56.5%)	1 (14.3%)	1 (20.0%)
Keep NE	6 (7.8%)	3 (7.1%)	3 (13.0%)	0	0
Gastrostomy/Jejunostomy	9 (11.7%)	8 (19.0%)	0	1 (14.3%)	0
Mortality	7 (9.1%)	2 (4.8%)	4 (17.4%)	1 (14.3%)	0
Loss follow-up	9 (11.7%)	8 (19.0%)	1 (4.3%)	0	0

*GERD, gastrointestinal reflux disease; UGI, upper gastrointestinal; OP, operation*.

* *Data present with mean ± SD*.

About 30 patients (40.0%) were weaned from the NE tube to an NG tube. Among these patients, 13 (43.3%) received the NE tube because of delayed gastric emptying, indicating the mostly transient gastric emptying dysfunction. Eight patients (10.4%) subsequently received surgical GT, and seven patients received Nissen fundoplication during the same surgery. The GT indications were recurrent emesis despite NE tube feeding (*N* = 7) and aspiration pneumonia (*N* = 1). Six patients continued receiving NE tube feeding and nine patients were lost to follow-up at the end of the study. Seven mortality cases (9.1%) were observed in the present study because of sepsis (*N* = 6) and uncal herniation (*N* = 1).

### Outcomes of Short- and Long-Term Placements of Nasoenteric Tubes

Short- (<8 weeks) and long-term placements of NE tubes are compared and summarized in Table 3. In the long-term group, most of the patients (79.3%) had neurologic dysfunction, and the baseline weight-for-age *z*-score and height-for-age *z*-score were lower than that of the short-term group. The NE tube feeding indications were not statistically different between the groups. Additional unexpected NE tube replacements were required in a long-term group (1.31 ± 1.93 vs. 0.17 ± 0.43, *p* < 0.001). Subsequent requirement of GT or jejunostomy was significantly higher in the long-term group than in the short-term group (24.1 vs. 4.2%, *p* = 0.008). The long-term group showed a higher increment of weight-for-age *z*-score after the NE tube feeding support.

**Table 3:** Short- (<8 weeks) and long-term (>8 weeks) use of NE tube feeding.

	**Short-term**	**Long-term**	***P*-value**
Number	48	29	
Age (years, mean ± SD)	3.72 ± 4.83	5.27 ± 6.23	0.958
Baseline weight-for-age z-score	−2.31 ± 2.41	−3.41 ± 3.04	0.088
Baseline height-for-age z-score	−2.13 ± 2.69	−2.89 ± 2.69	0.243
**NE tube placement**			
Insertion duration	26.15 ± 11.86	311.83 ± 386.21	^*^<0.001
Average insertion times/person	1.19 ± 0.45	4.10 ± 4.82	^*^<0.001
Unexpected times	0.17 ± 0.43	1.31 ± 1.93	^*^<0.001
**Comorbidity**			
Neurologic	26 (54.1%)	23(79.3%)	^*^0.026
Preterm	15 (31.2%)	10 (34.4%)	0.769
**NE tube indications**			
Severe GERD	26 (54.2%)	16 (55.2%)	0.932
Delayed gastric emptying	12 (25%)	11 (37.9%)	0.230
Postsurgery nutrition support	5 (10.4%)	0	0.072
Partial UGI obstruction	5 (10.4%)	2 (6.9%)	0.603
**Growth**			
Weight-for-age z-score alternations	0.11 ± 0.85	0.21 ± 1.85	0.782
Height-for-age z-score alternations	−0.12 ± 0.56	−0.50 ± 1.88	0.301
**Outcomes**			
Keep NE tube feeding	3 (6.3%)	3 (10.3%)	0.516
Oral feeding	15 (31.3%)	1 (3.4%)	^*^0.004
NG tube feeding	19 (39.6%)	11 (37.9%)	0.885
Gastrostomy/Jejunostomy	2 (4.2%)	7 (24.1%)	^*^0.008
Mortality	3 (6.3%)	4 (13.8%)	0.265
Loss follow-up	6 (12.5%)	3 (10.3%)	0.775

*SD, standard deviation; GERD, gastroesophageal reflux disease; UGI, upper gastrointestinal; NE, Nasoenteric tube; NG, Nasogastric tube*.

* *Numerical data were analyzed using the Student's t-test, and categorical data were analyzed using the Chi-squared test. A p < 0.05 was considered to be statistically significant*.

### Nasoenteric Tube-Related Adverse Events

A total of 47 unexpected reinsertions (26.7%) were performed due to tube dislodgement (*N* = 37, 78.7%), tube dysfunction (*N* = 9, 19.1%), and tube breaks (*N* = 1, 0.02%). No tube-related bacterial enterocolitis, intussusception, and refeeding syndrome were observed. One major NE tube-related complication was observed in a 2-month-old girl with congenital pulmonary lymphangiectasia. Bowel perforation was found at 4 cm distal to the ligament of Treitz after NE tube placement for 6 days. The patient expired 32 days later because of ventilator-associated pneumonia. No direct NE tube-related mortality was observed.

## Discussion

This research showed positive clinical outcomes from different aspects for pediatric patients requiring enteral nutrition therapy. Endoscopic-guided NE tube placement is relatively safe, and a high success rate procedure, for pediatric patients.

Children who require tube feeding mostly have multiple medical diagnoses, of which congenital abnormalities (42%), perinatal problems (38%), and neurologic diseases (16%) are the most common comorbidities, as indicated in the Netherlands study (14). Another study in Poland reported neurological disorders (64.2%) as the most common underlying diseases indicated for tube feeding (15). Neurologic dysfunction, prematurity, and congenital anomalies accounted for 63.6, 32.5, and 18.2%, respectively, of comorbidities in patients of this study. There were 12 cases (15.6%) of underlying diseases of prematurity and neurologic dysfunction, and, in this case, tube feeding can improve the nutritional status, drooling, secretion management, and constipation and ease caregiver medication administration and feeding (16).

The procedure of NE tube insertion in this study was safe without complications except for placement failure twice. The success rate of using endoscopic-guided enteric tube was 98.9% in our institution, similar to that in the previous reports (5, 17). The most common complication was tube dislodgement for 37 times, which occurred in 19 patients (24.7%). The incidence of accidental feeding tube dislodgement ~28.9–40% in the previous reports (18, 19). Tube occlusion episodes totaled nine times in eight patients. Inadequate flushing and administration of more than three kinds of medications may contribute to tube occlusion. Enteral devices into the small bowel had been reported with serious adverse events, including bowel perforation, volvulus, major bleeds, and intussusceptions (20, 21). None of the patients exhibited serious adverse events, except for one (1.3%) who had developed bowel perforation at 6 days post-insertion. No tube-related mortality was observed.

In the present study, the most common indication for NE tube placement is severe GERD (54.5%), and, overall, 71.4% of the patients who exhibited improved GERD-symptoms (vomiting, desaturations/bradycardia during/after feeding) after NE tube feeding. Small bowel feeding reduces the risk of aspiration pneumonia in mechanically-ventilated adult patients (22, 23) although evidence in pediatric patients is equivocal (24, 25). NE tube feeding can reduce aspiration pneumonia episodes in patients of this study.

Delayed gastric emptying, a secondary underlying disease, or sedatives or muscle relaxants can increase the risk of aspiration pneumonia and interrupt enteral feeding (26). More than 50% of critically ill children can present a high gastric residual volume (26). The European Society for Pediatric Gastroenterology, Hepatology, and Nutrition (ESPGHAN) of the expert group recommends that transpyloric tube feeding may be considered to provide enteral nutrition when gastric feeding fails in critically ill children (6). Nguyen et al. suggested that the first step for treating feeding intolerance in critically ill patients should be the use of prokinetics, such as erythromycin and metoclopramide. If the prokinetic therapy is unsatisfactory, post-pyloric feeding should be considered (27). The use of prokinetics, including metoclopramide and mosapride, had been attempted on patients but with an insufficient response. Thus, post-pyloric tube feeding was initiated. As a result, the average daily feeding volume significantly increased from 144.8 ± 136.5 to 1,103.1 ± 524.7 ml in the delayed gastric emptying group. The feeding volume increment could help to wean from PN support, reduce further septic complications, and reduce the hospitalization days.

Four out of five post-surgery patients were from the burn injury population, and the remaining one had necrotizing fasciitis. Pediatric patients with burns required high levels of nutrition due to body growth and development, high levels of oxidative stress, an intense inflammatory response, and prolonged hypercatabolism (28). The small bowel is the first portion of the GI tract that regains the function of absorption and motility within 6–8 h post-operation. Nutrition support strategies are suggested as early as possible if enteral feeding shows no contraindication and can be started in a safe manner *via* the nasoduodenal tube or the nasojejunal tube until sufficient orally ingested calories are obtained (29). Patients received NE tube insertion within 24–48 h after surgery, with an insertion duration average of 27.2 ± 4.0 days. The albumin level significantly increased from 2.68 ± 0.67 to 3.66 ± 0.33 g/dl during this period. A meta-analysis and systemic review study reported that early enteral nutrition provided within 24 h after injury showed benefits of a reduced duration of hospital stay and low caloric deficit and weight loss but a high incidence of diarrhea and vomiting in pediatric burn patients (30). Hypoalbuminemia in burn patients is strongly associated with burn severity and high mortality rate (31, 32).

Our study showed positive nutritional outcomes, including a statistically significant increase in the daily feeding amount and serum albumin level, after NE tube feeding for an average of 133.8 ± 272.9 days. Patients with weight-for-age *z*-score and height-for-age *z*-score < −3 accounted for 45.5 and 26.0%, respectively, indicating that patients were severely malnourished at the baseline. We further stratified patients into three different baseline weight-for-age *z*-score for evaluation and found that NE tube feeding may help in better improving the weight-for-age *z*-score in the baseline malnourished patients. An appropriate linear growth was not achieved in our study. Compared with other GT (33), percutaneous endoscopic GT (PEG) (34), and the surgical jejunostomy (SJ) studies (35), our patients had similar weight-for-age *z*-score improvement from −4.02 to −3.42 in 1 year in NE tube feeding, from −2.8 to −1.8 in 1 year in GT, from −1.5 to 0.9 in 6 months in PEG, and from −3.7 to −2.6 in 1 year in SJ. In addition, we analyzed the growth of patients who subsequently received surgical GT (*N* = 8) and fundoplication (*N* = 7) and showed a decrease in weight-for-age *z*-score from −2.76 ± 1.73 to −3.25 ± 2.91 and an increase in height-for-age z-score from −3.34 ± 2.35 to −3.19 ± 2.60 during follow-up for 1,164.3 ± 697.7 days.

The strengths of this study include a relatively large sample size and the standardization of enteral nutrition management and consultation for surgical approaches, which are based on the guidelines for pediatric gastroenterologists and pediatric surgeons. In addition, this study statistically evaluated factors related to favorable or unfavorable nutritional outcomes in NE tube feeding; such an evaluation was never discussed in previous literature studies. This study has several limitations. First, this work is a retrospective review with an inherent difference in the timing of initiation of NE tube feeding among patients with different underlying diseases. Second, nutritional parameters (serum Hb and albumin) were not checked in several patients because they achieved clinical success. Finally, we did not compare the differences in the nutritional outcomes between patients with long-term NE tube feeding and those with enterostomy.

## Conclusions

Endoscopic-guided placement of NE tube is a relatively safe, non-invasive procedure for pediatric patients who require enteral nutrition therapy but are intolerable to gastric feeding. The NE tube feeding intervention can significantly increase feeding volumes, reduce clinical symptoms, wean from PN support, and improve growth conditions, even in the baseline severely malnourished patients. NE tube feeding may keep the weight gain steady without any significant adverse event in a long-term use.

## Data Availability Statement

The raw data supporting the conclusions of this article will be made available by the authors, without undue reservation.

## Ethics Statement

The studies involving human participants were reviewed and approved by Ethics Committee of Chang Gung Memorial Hospital (Ref. 202001377B0). Written informed consent from the participants' legal guardian/next of kin was not required to participate in this study in accordance with the national legislation and the institutional requirements.

## Author Contributions

C-CC, H-CC, M-WL, and P-JY: diagnosis and management of the patients. M-CC and H-CC: writing of the manuscript, data analysis and interpretation, and final approval of the manuscript. H-CC: critical evaluation and revision of the manuscript. All authors contributed to the article and approved the submitted version.

## Conflict of Interest

The authors declare that the research was conducted in the absence of any commercial or financial relationships that could be construed as a potential conflict of interest.
